# Decreased Serologic Response in Vaccinated Military Recruits during 2011 Correspond to Genetic Drift in Concurrent Circulating Pandemic A/H1N1 Viruses

**DOI:** 10.1371/journal.pone.0034581

**Published:** 2012-04-13

**Authors:** Dennis J. Faix, Anthony W. Hawksworth, Christopher A. Myers, Christian J. Hansen, Ryan G. Ortiguerra, Rebecca Halpin, David Wentworth, Laura A. Pacha, Erica G. Schwartz, Shawn M. S. Garcia, Angelia A. Eick-Cost, Christopher D. Clagett, Surender Khurana, Hana Golding, Patrick J. Blair

**Affiliations:** 1 Department of Operational Infectious Diseases, Naval Health Research Center, San Diego, California, United States of America; 2 Viral Programs, J. Craig Venter Institute, Rockville, Maryland, United States of America; 3 Disease Epidemiology Program, Army Public Health Command, Aberdeen Proving Ground, Maryland, United States of America; 4 Operational Medicine, U.S. Coast Guard Headquarters, Washington, D. C., United States of America; 5 Preventative Medicine, Naval Hospital Beaufort, Beaufort, South Carolina, United States of America; 6 Division of Epidemiology and Analysis, Armed Forces Health Surveillance Center, Silver Spring, Maryland, United States of America; 7 Preventative Medicine, Navy and Marine Corps Public Health Center, Portsmouth, Virginia, United States of America; 8 Division of Viral Products, Center for Biologics Evaluation and Research, Food and Drug Administration, Bethesda, Maryland, United States of America; University of Texas Medical Branch, United States of America

## Abstract

**Background:**

Population-based febrile respiratory illness surveillance conducted by the Department of Defense contributes to an estimate of vaccine effectiveness. Between January and March 2011, 64 cases of 2009 A/H1N1 (pH1N1), including one fatality, were confirmed in immunized recruits at Fort Jackson, South Carolina, suggesting insufficient efficacy for the pH1N1 component of the live attenuated influenza vaccine (LAIV).

**Methodology/Principal Findings:**

To test serologic protection, serum samples were collected at least 30 days post-vaccination from recruits at Fort Jackson (LAIV), Parris Island (LAIV and trivalent inactivated vaccine [TIV]) at Cape May, New Jersey (TIV) and responses measured against pre-vaccination sera. A subset of 78 LAIV and 64 TIV sera pairs from recruits who reported neither influenza vaccination in the prior year nor fever during training were tested by microneutralization (MN) and hemagglutination inhibition (HI) assays. MN results demonstrated that seroconversion in paired sera was greater in those who received TIV versus LAIV (74% and 37%). Additionally, the fold change associated with TIV vaccination was significantly different between circulating (2011) versus the vaccine strain (2009) of pH1N1 viruses (ANOVA *p* value = 0.0006). HI analyses revealed similar trends. Surface plasmon resonance (SPR) analysis revealed that the quantity, IgG/IgM ratios, and affinity of anti-HA antibodies were significantly greater in TIV vaccinees. Finally, sequence analysis of the HA1 gene in concurrent circulating 2011 pH1N1 isolates from Fort Jackson exhibited modest amino acid divergence from the vaccine strain.

**Conclusions/Significance:**

Among military recruits in 2011, serum antibody response differed by vaccine type (LAIV vs. TIV) and pH1N1 virus year (2009 vs. 2011). We hypothesize that antigen drift in circulating pH1N1 viruses contributed to reduce vaccine effectiveness at Fort Jackson. Our findings have wider implications regarding vaccine protection from circulating pH1N1 viruses in 2011–2012.

## Introduction

Crowded living quarters and stress can increase the potential for respiratory infections among military service members and lead to respiratory disease outbreaks [1], [2], [3], [4]. Military members are particularly susceptible to epidemics from seasonal or novel influenza viruses, such as in 1918 when the rapid spread of A/H1N1 among deploying troops and recruits resulted in an attack rate estimated at 20% to 40% of U.S. Army and Navy personnel [5]. In 1976, A/H1N1 “swine influenza” infections in soldiers stationed at Fort Dix, New Jersey [6], drove fears of a pandemic and recommendations of widespread vaccination of the U.S. population [7]. The emergence of a quadruple reassorted A/H1N1 virus (pH1N1) on the U.S.–Mexico border in 2009 [8], [9], [10] resulted in a pandemic that stressed medical capacity and hampered military operations.

Since 1996, the Department of Defense (DoD) has conducted population-based febrile respiratory illness (FRI) surveillance at military recruit training centers (RTCs) across the United States [11], [12]. The DoD continually monitors influenza vaccine effectiveness, one purpose of which is to elucidate factors contributing to vaccine failure [13]. This representative sampling of febrile recruits allows for an estimate of disease burden, responsible pathogens, and pathogen subtypes. To counter outbreaks of influenza, the trivalent inactivated vaccine (TIV) has been used to protect military service members over the last 60 years [14]. Due to the ease of administration and often earlier availability, the live attenuated influenza vaccine (LAIV) has been preferentially utilized by the DoD since 2003 [15], especially among recruit populations. Because military recruits are universally vaccinated prior to the first week of training, cases occurring past the first 2 weeks of training, when vaccine-induced immunity is established in a healthy population, may be an indication of decreased vaccine effectiveness.

In the early months of 2011, FRI surveillance evidenced a sharp rise in pH1N1 cases among LAIV-vaccinated recruits after the second week of training at the U.S. Army RTC at Fort Jackson, South Carolina, suggesting reduced effectiveness for the pH1N1 component [16]. During this outbreak, one vaccinated recruit was hospitalized and died following laboratory-confirmed pH1N1 infection. To understand the contributing factors resulting in increased rates of pH1N1, we undertook a serological study to describe the corresponding antibody responses. Sera were drawn 4–5 weeks post-vaccination from recruits at Fort Jackson, Columbia, South Carolina (Fort Jackson), Marine Corps Recruit Depot, Parris Island, South Carolina (MCRD-PI), and Coast Guard Training Center, Cape May, New Jersey (Cape May) in March 2011. Microneutralization (MN) and hemagglutination inhibition (HI) tests were conducted using standardized reagents in the 2010–2011 World Health Organization (WHO) Influenza Reagent Kit [17]. To study response to the 2011 circulating pH1N1 strain, ferret antisera were generated from a pH1N1 virus (A/CA/17/2011 H1N1) isolated from a recruit at Fort Jackson in January 2011. Contemporaneous isolates from recruit training sites across the United States were sequenced and analyzed for divergence in the hemagglutination gene (HA). Herein we show that the level and affinity of serum antibodies generated in response to influenza vaccination in our population varied by vaccine type (TIV vs. LAIV) and had significantly different specificity to locally circulating pH1N1 viruses compared with the vaccine strain. Decreased serologic response corresponded to modest antigenic drift in the HA gene of pandemic A/H1N1 viruses circulating in the region in 2011.

## Results

### Influenza Vaccination, Sera Collection, and Study Population Demographics

All recruits at Fort Jackson and male recruits at MCRD-PI received the 2010–2011 LAIV, while female recruits at MCRD-PI were vaccinated with TIV, as required by local protocol. At Cape May, recruits were vaccinated with TIV.

Population-based FRI surveillance conducted between December 2010 and March 2011 noted laboratory-confirmed influenza rates (per 100-person weeks) among influenza-vaccinated military recruits at the three RTCs as: 0.15, Fort Jackson; 0.06, MCRD-PI; and 0.05, Cape May. During this time, influenza A/H1N1 2009, A/H3N2, and B viruses circulated in CDC region 4, with A/H1N1 2009 virus predominating (Centers for Disease Control and Prevention [CDC] FluView. 2010–2011 influenza season week 10; http://www.cdc.gov/flu/weekly/weeklyarchives2010-2011/weekly10.htm). Among 240 sampled FRI cases at Fort Jackson between January and March of 2011, there were 64 pH1N1, 3 A/H3N2 and 5 influenza B viruses determined by reverse transcriptase polymerase chain reaction (RT-PCR) analysis. Influenza infections continued at the study sites through March.

In this study, a total of 540 trainees were enrolled including 201 from Fort Jackson, 259 from MCRD-PI, and 80 from Cape May. The average recruit numbers at the respective sites during this period were 9000 at Fort Jackson, 4000 at MCRD–PI, and 550 at Cape May. From 260 participants who reported no fever during the preceding 4–5 weeks of basic training and no influenza vaccination the previous year, a subset of 142 serum pairs were selected for serologic testing. These included the first 50 enrollees from Fort Jackson and MCRD-PI, and 42 from Cape May who met the aforementioned criteria. Baseline sera were obtained from the Department of Defense Serum Repository (DoDSR) in Silver Spring, MD. Post-vaccination sera were compared with matched baseline (pre-vaccination) samples drawn on average 133 days pre-vaccination (range 2–498 days). The mean ages of study participants were 22.1 years for the LAIV group and 20.9 years for the TIV recruits (*p* = 0.054). Sex varied, with 83% men in the LAIV recipient group versus 59% men in the TIV group (*p* = 0.001). The mean days post-vaccination for sera used in the study were 35.7 days in the LAIV group and 32.6 days in the TIV group (*p*<0.0001).

### Seroresponse as a Correlate of Microneutralization Titer

Samples from the three RTC sites were compared within TIV and LAIV-vaccinated groups. Among the paired sera studied, 78 were from LAIV-vaccinated individuals and 64 from TIV-vaccinated recruits. Madin-Darby canine kidney (MDCK) cell-generated influenza viruses A/CA/7/2009 H1N1 (2009 pH1N1), A/Perth/16/2009 H3N2 (H3N2), and A/CA/17/2011 H1N1 (2011 pH1N1) titered to a consistent concentration that gave 75% cytopathic effect (CPE) were utilized in the MN assays.

**Figure 1:**
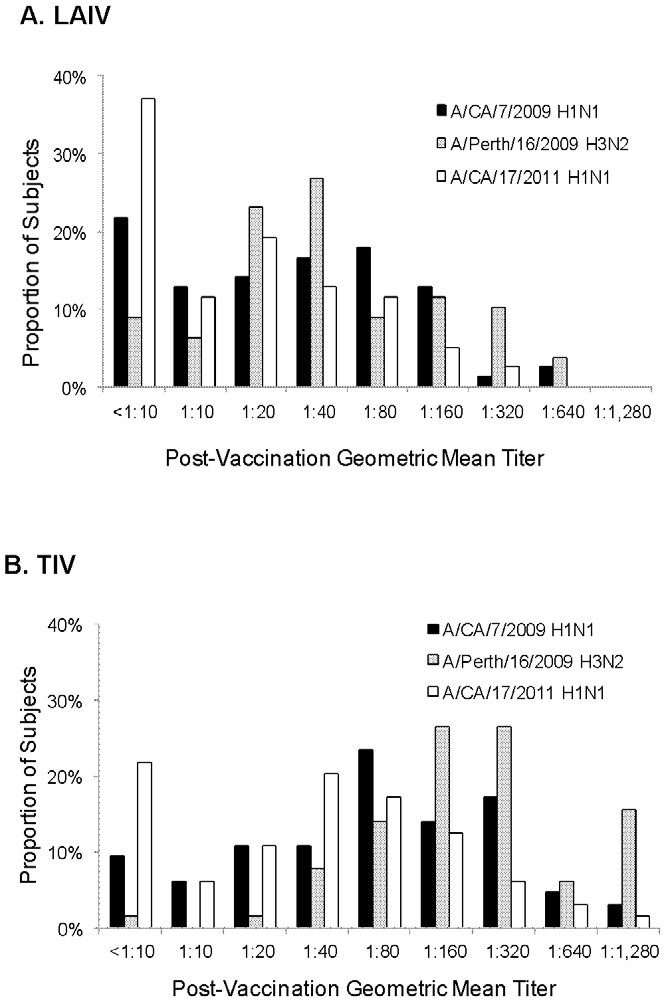
Post-vaccination microneutralization titer distribution responses in vaccinated recruits based upon vaccine type.

The proportions of LAIV and TIV vaccinees for the range of post-vaccination titers were plotted for the vaccine strain H3N2 and 2009 pH1N1 viruses and the circulating 2011 pH1N1 virus (Figure 1). Overall, titers in TIV vaccinees were greater than LAIV titers. Also, responses against the 2011 pH1N1 virus were decreased compared with 2009 pH1N1 and H3N2 in both the LAIV and TIV groups. Serologic responses in vaccinated recruits following MN analysis are summarized in Tables 1 and 2. The pre-vaccine geometric mean titer (GMT) levels among the groups were not significantly different, although the percentage of individuals with titers ≥40 was markedly higher in both 2009 pH1N1 and H3N2 groups likely because these viruses had circulated for longer than the 2011 pH1N1 virus. More individuals in both vaccine groups exhibited seroresponse against H3N2 than the 2009 and 2011 pH1N1 viruses. Post-vaccine seroprotection among LAIV vaccinees for 2009 pH1N1, H3N2, and 2011 pH1N1 was 51%, 62%, and 32%, while for TIV the percentages were 73%, 97%, and 61%, respectively (Tables 1 and 2). Among naïve subjects (those with initial titers ≤40), seroconversion was greater among TIV than LAIV recipients for H3N2 (98% vs. 57%; *p*<0.0001), 2009 pH1N1 (74% vs. 43%; *p* = 0.0007), and 2011 pH1N1 (64% vs. 30%; *p*<0.0001), and for any virus (77% vs. 43%; *p*<0.0001) (data not shown).

**Table 1:** Serologic responses in vaccinated recruits as measured by microneutralization assay, LAIV-vaccinated recruits (n = 78).

Virus	A/CA/7/2009 H1N1	A/Perth/16/2009 H3N2	A/CA/17/2011 H1N1
# Pre-vac. titer ≥40 (%)	17 (22%)	17 (22%)	8 (10%)
GMT Pre-vac. (95% CI)	10.5 (8.4–13.1)	12.5 (10.0–15.7)	7.8 (6.3–9.8)
# Post-vac. titer ≥40 (%)	40 (51%)	48 (62%)	25 (32%)
GMT post-vac. (95% CI)	30.9 (23.1–41.4)	47.2 (35.2–63.1)	17.7 (1323.7)
Seroconversion (%)^1^	27 (35%)	37 (47%)	22 (28%)
Fold change (95% CI)^2^	2.2 (1.7–2.9)	2.8 (2.1–3.7)	1.5 (1.1–2.0)

CI, confidence interval; GMT, geometric mean titer; LAIV, live attenuated influenza vaccine.

1 Seroconversion is defined as a 4-fold increase in titer from pre-vaccine to post-vaccine titer.

2 Fold change adjusted for pre-vaccine seroprotection levels.

**Table 2:** Serologic responses in vaccinated recruits as measured by microneutralization assay, TIV-vaccinated recruits (n = 64).

Virus	A/CA/7/2009 H1N1	A/Perth/16/2009 H3N2	A/CA/17/2011 H1N1
# Pre-vac. titer ≥40 (%)	10 (16%)	19 (30%)	5 (8%)
GMT pre-vac. (95% CI)	9.7 (7.6–12.4)	18.0 (14.0–23.0)	7.4 (5.8–9.5)
# Post-vac. titer≥40 (%)	47 (73%)	62 (97%)	39 (61%)
GMT post-vac. (95% CI)	77.9 (56.5–107.4)	227.5 (165.0–313.8)	39.4 (28.5–54.3)
Seroconversion (%)^1^	47 (73%)	54 (84%)	41 (64%)
Fold change (95% CI)**	5.6 (4.1–7.6)	10.2 (7.6–13.8)	3.4 (2.5–4.7)

CI, confidence interval; GMT, geometric mean titer; TIV, trivalent inactivated influenza vaccine.

1 Seroconversion is defined as a 4-fold increase in titer from pre-vaccine to post-vaccine titer.

** Fold-change adjusted for pre-vaccine seroprotection.

**Figure 2:**
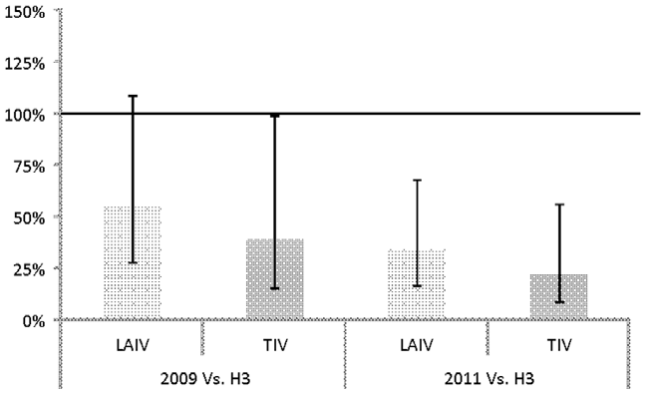
Estimate of odds of a 4-fold conversion relative to conversions against the H3N2 virus.

The baseline titer-adjusted fold change in MN titer associated with TIV vaccination was significantly different between the 2011 and 2009 pH1N1 viruses. To illustrate these differences, we modeled the logistic regression odds of a 4-fold conversion against 2011 and 2009 pH1N1 relative to conversion against the H3N2 strain (Figure 2). Results demonstrated that in TIV- and LAIV-vaccinated recruits the adjusted odds of a 4-fold rise in titer were sharply lower in the 2011 pH1N1 virus than the H3N2 virus (analysis of variance (ANOVA) *p* = 0.0006). Finally, while the proportion of individuals with pre-vaccine serum antibody titers (≥20) for all three viruses was higher by HA assays, the overall trends were similar to those seen with the MN data.

### TIV- and LAIV-Elicited 2009-pH1N1 HA1-Specific Antibodies with Different Isotype Profiles and Binding Avidity

**Figure 3:**
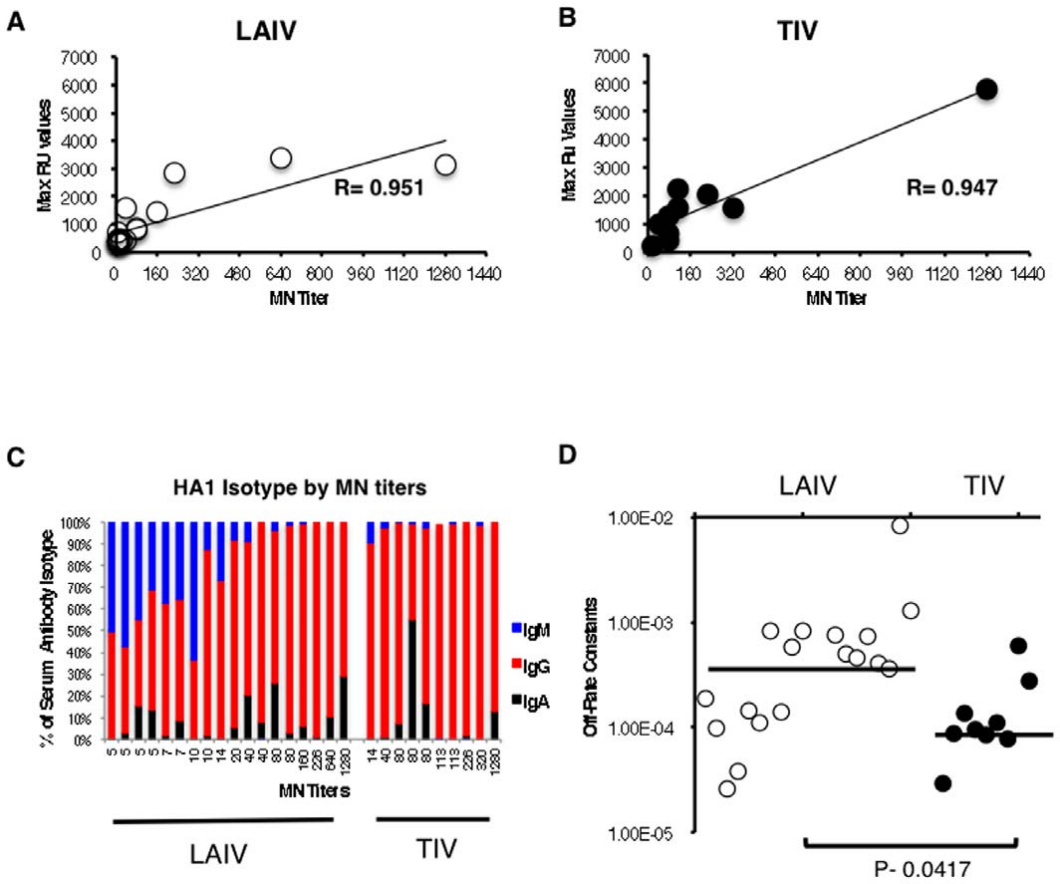
Affinity measurements isotyping and total anti-HA1 binding antibody in human sera following immunization. **(A–B) Correlation between in vitro MN titers and rHA1 binding in human sera following immunization with H1N1 vaccine in TIV and LAIV groups.** Steady-state equilibrium analysis of the binding of vaccine serum IgG to properly folded functional HA1 oligomers was measured using surface plasmon resonance (SPR). Ten-fold diluted post-H1N1 vaccination sera from vaccine groups (LAIV in A, TIV in B) were injected simultaneously onto HA1 immobilized on a sensor chip, free of peptide. Binding was recorded in resonance units (RU) values. The maximum RU values for HA1 binding by serum antibodies obtained from vaccinated individuals with either LIAV (A) or TIV (B) vaccination is shown on the Y-axis. The MN titer is expressed as end-point neutralizing antibody titer of post-H1N1 vaccine sera and is depicted on the X-axis. **(C) The isotype of serum antibodies bound to rHA1 for the two vaccine groups.** Data shown are the means for serum from two independent experiments. **(D) Antibody avidity measurements in polyclonal serum by off-rate constants using SPR.** Antibody off-rate constants, which describe the stability of the complex were determined directly from the serum/plasma sample interaction with rHA1 protein using SPR in the dissociation phase. For accurate measurements, parallel lines in the dissociation phase for the 10-fold and 100-fold dilution for each post-vaccination human sera were required. The off-rate constants were determined from two independent SPR runs. SPR analysis of post-vaccinated human sera with LAIV (left) or TIV (right) from the vaccine trial was performed with properly folded H1N1pdm09 HA1 (A/CA/7/2009) [60]. Serum antibody off-rate constants for vaccinees (each symbol is one individual) were plotted. Correlation statistics of affinity measurement and off-rate constants of sera binding to rHA1 between LAIV and TIV vaccinees were statistically significant with *p*<0.05 (T-test).

Sera samples from a randomly selected subset of TIV (n = 10) and LAIV (n = 18) vaccinees were analyzed by surface plasmon resonance (SPR) for post-vaccination serum antibody binding to properly folded, functional oligomeric recombinant HA1 peptide against the 2009 pH1N1 strain. The maximum resonance unit (max RU) values for the serum antibody binding antibodies to rHA1 from LAIV (Figure 3A) and TIV (Figure 3B) vaccinated groups strongly correlated with the MN titers against the pH1N1 vaccine strain A/CA/7/2009. Isotyping of post-vaccination antibodies demonstrated that the majority (>90%) of the binding to the HA1 globular domain (1–330 amino acids) in the TIV-vaccinated individuals was mediated by IgG antibodies, with a small contribution (≤10%) from IgA antibodies irrespective of the MN titers. In contrast, in the LAIV-vaccinated individuals, a significant amount of rHA1 binding antibodies were IgM, especially in sera with lower MN titers to 2009 pH1N1 (Figure 3C).

### TIV-Vaccinated Individuals Show Better Antibody Affinity Maturation to pH1N1 Compared with LAIV-Vaccinated Individuals

To further evaluate the quality of the antibodies elicited after vaccination, antibody affinity maturation as measured by SPR were utilized to calculate the antibody dissociation off-rates for individual sera in TIV- and LAIV-vaccinated subjects (Figure 3D). Antibody off-rate constants, which describe the stability of antigen-antibody complexes, were determined directly from serum sample interaction with HA1 globular domain using SPR in the dissociation phase as described [18]. The antibody dissociation rates for the post-vaccination serum to the 2009-pH1N1-rHA1 averaged 7×10^−4^ per second (ranged between 8.31×10^−3^ and 2.56×10^−5^) for the individuals following LAIV vaccination (Figure 3D, open circles). In contrast, TIV vaccination resulted in significantly lower anti-HA1 antibody dissociation rates or higher antibody affinity (Figure 3D, filled circles) that averaged 8.76×10^−5^ per second (ranged between 5.94×10^−4^ and 2.92×10^−5^). The mean antibody off-rates to 2009-pH1N1-HA1 were significantly different between TIV- and LAIV-vaccinated individuals (*p* = 0.0417) (Figure 3D).

### Sequencing and Phylogenetic Analysis of Isolated Viruses

**Figure 4:**
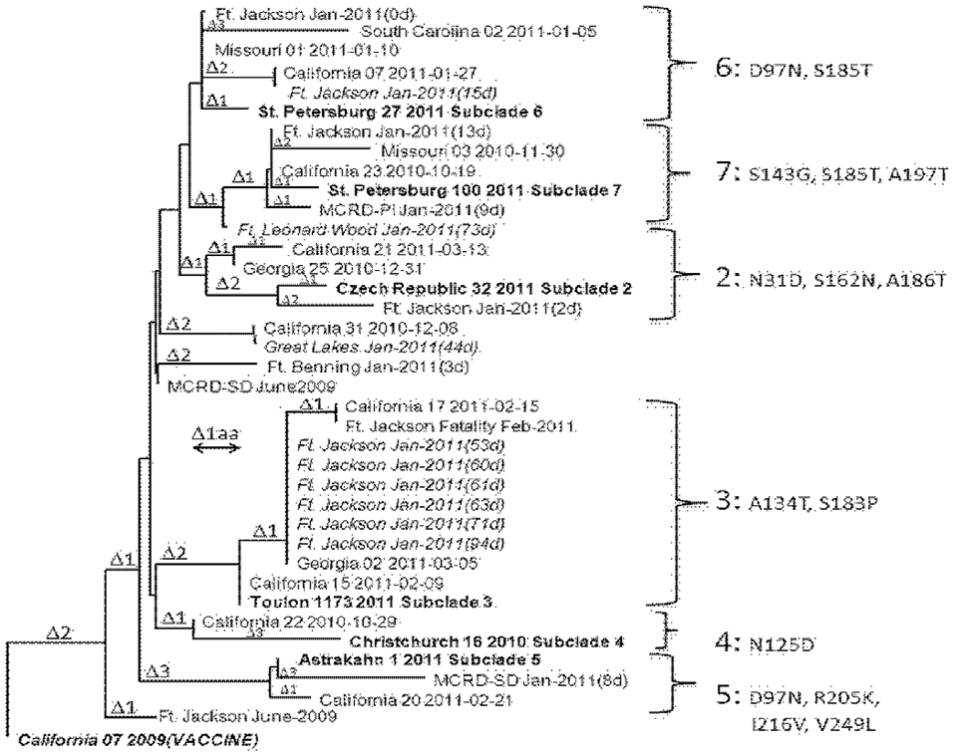
Phylogenetic analysis of the hemagglutinin (HA) gene of influenza pH1N1 isolates. The phylogenetic tree was constructed using the neighbor-joining method (CLUSTAL W method) of the MegAlign program (Lasergene software suite, DNASTAR Inc., Madison, WI). The tree represents amino acids 111 through 360 of the mature HA protein. Reference strains for the six circulating subclades (2–7) are shown in bold text, with their defining mutations shown to the right. The vaccine strain, A/CA/7/2009, is shown in bold and italicized. Isolates collected by NHRC from recruits who had been vaccinated greater than 14 days are italicized. Strains from the same geographical location and time of collection from the GISAID database are included in the tree as well as two samples collected from the same recruit training sites in 2009. The scale indicates the distance created by a one amino-acid difference between sequences. The number of amino acid changes between subclades is denoted by a delta symbol (Δ).

To examine whether decreased serologic response from the A/CA/7/2009 H1N1 vaccine strain to circulating pH1N1 viruses isolated from recruits training in the southeastern United States in early 2011 correlated with antigenic drift, the HA1 genes from previous circulating and contemporaneous representative viruses were sequenced and a phylogenetic tree constructed using the neighbor-joining method (CLUSTAL W) of the MegAlign program (Lasergene software suite; DNASTAR Inc., Madison, WI) (Figure 4). Viruses in the tree are represented by amino acids 112 through 360 of the mature HA protein. Six circulating subclades of 2009 pH1N1 (Groups 2–7) have been defined previously http://www.ecdc.europa.eu/en/publications/Publications/1110_SUR_Influenza_virus_characterization_August_September%202011.pdf). Included in the phylogenic tree were 10 viruses isolated from Fort Jackson between January and March 2011, as well as representative viruses from three recruits at Fort Benning, Cape May, and MCRD-PI collected concurrently. These include Great Lakes RTC Jan-2011 (CY092872.1); Marine Corps San Diego Jan-2011 (CY092880.1); Fort Jackson Jan 2011 (CY092888.1); Marine Corps Parris Island Jan-2011 (CY092896.1); Fort Benning Jan-2011 (CY092904.1); Mexico InDRE1947 2011 (CY089391.1); and Mexico InDRE1945 2011 (CY089387.1). Viruses isolated during the January 2011 outbreak at Fort Jackson are italicized. For reference, the tree contained representative sequences from Fort Jackson and MCRD San Diego collected during 2009, the vaccine strain, A/CA/7/2009, and, finally, reference strains for the six circulating subclades of 2009 pH1N1 (Groups 2–7) that have been defined by specific mutations. Phylogenetic analysis of viruses collected from recruits during 2010–2011 demonstrated the presence of five of the six previously characterized groups of 2009 pH1N1 viruses. Viruses isolated between January and March from Fort Jackson recruits vaccinated greater than 2 weeks clustered into Group 3 (Figure 4). Relative to the vaccine strain, Group 3 viruses have four mutations across the HA coding region sequenced, including S183P. Interestingly, viruses collected from a Fort Jackson recruit who had been vaccinated only 2 days prior to illness branched into Group 2. Representative viruses from recruits in 2011 segregated into Groups 2, 6, and 7. These were detected contemporaneously to the outbreak at Fort Jackson but were not associated with the January outbreak and did not circulate widely. Whole genome analysis of a representative strain (Fort Jackson, January 2011) had seven mutations from the vaccine strain (data not shown). Three of these mutations, P83S, S203T, and I321V occurred very early during the evolution of the pandemic virus and were fixed in the 2009 pH1N1 population. Two of these mutations (A134T and S183P) are represented in the Group 3 subclade.

## Discussion

A survey to measure seroresponses to seasonal influenza vaccine was conducted among military recruits at three RTCs in the eastern and southeastern United States March 2011, following intense transmission of pH1N1 in January, including one death, among vaccinated recruits in South Carolina [16]. Serum antibody studies indicated that (1) compared with LAIV, TIV induced greater total serum antibodies, a more mature antibody response as measured by isotype distribution, and antibodies with greater affinity to HA1; (2) both LAIV and TIV induced seroresponses against a circulating 2011 pH1N1 clade, but responses were significantly lower than those against the vaccine 2009 pH1N1 strain; and (3) HA1 sequence analysis from concurrently circulating pH1N1 strains in 2011 demonstrated that a single clade with moderate drift from the pH1N1 vaccine strain was responsible for the outbreak. We hypothesize that increased pH1N1 infection among vaccinated recruits at Fort Jackson in early 2011 resulted from antigenic mismatch between the circulating pH1N1 strain and the pH1N1 vaccine strain.

Military recruits are homogeneously healthy and young. As a result of crowding and a challenging training environment, recruits experience higher respiratory disease rates than non-recruits [19]. Vaccination reduces influenza rates in military populations providing that the vaccine is a good antigenic match [20], [21], [22]. Ongoing syndromic and laboratory-based surveillance provide annual influenza vaccine effectiveness estimates [23]. Vaccine effectiveness is a function of the antigenic match between circulating and vaccine influenza strains and host factors such as previous influenza exposure, age, and immune status [24], [25], [26], [27], [28], [29], [30]. Recruits generally receive multiple, simultaneous vaccinations during the first days at the RTC. These factors must be taken into account when considering vaccine response and effectiveness data.

Both TIV and LAIV have been shown to be effective in children and adults, although multiple studies in children aged 6 months to 18 years have demonstrated that LAIV provides a greater seroresponse than TIV [25], [26], [31]. While results in adults have been mixed [32], TIV vaccination has generally been reported as more effective than LAIV [33]. When vaccines are staggered in a recruit setting rather than given simultaneously, clinic visits for respiratory disease decreased by up to 20% [34]. A large, cross-sectional study during the 2005–2006 influenza season among military personnel determined that LAIV vaccination provided greater protection from laboratory-confirmed influenza than the TIV [15]. In non-recruits TIV provided greater protection. Over the 2007–2008 influenza season, TIV was found to be 50% more effective than LAIV in adult populations [24]. A monovalent LAIV pH1N1 vaccine showed differing vaccine efficacy based upon age groups when influenza-like symptoms were considered [35]. In our study, geographic differences (vaccine type and circulating strain) and ongoing adenovirus transmission at the RTC precluded a clear comparison between vaccine types based on symptom data alone.

Our results show differential immune response between LAIV and TIV consistent with previous reports. The quantitative and qualitative robustness of T and B cell memory responses to viral antigens are mediated by previous exposure and/or vaccination. Recently, a study in children ages 6–35 months found that LAIV conferred broader heterotypic αβ and γδ T cell immunity against conserved influenza peptides than TIV [36]. He et al. determined that the phenotypic changes of influenza-specific CD8^+^ differed significantly between LAIV and TIV depending on the age of the vaccinee. The authors of this study speculated that the route of vaccination influenced antigenic presentation [37]. A prospective, randomized trial to compare the safety and efficacy of LAIV and TIV in adults in South Africa found that those ≥60 years old had better T cell responses to LAIV but superior humoral immune responses to TIV [38]. In our work, TIV vaccinated recruits exhibited increased antibody class switching (IgM->IgG) and affinity maturation to 2009 pH1N1 compared with LAIV-vaccinated individuals. While serum antibody HI titers are a correlate of protection, modest antibody titers following LAIV vaccination do not necessarily indicate a failure of protection [39]. However, the generation of anti-influenza antibodies with increased affinity among the TIV vaccinees likely allowed more effective clearance of infecting influenza viruses. Differential presentation in the two vaccines of similar antigens might have driven alternate immunoglobulin class switching and maturation routes. SPR on post-vaccination antibody binding to HA1 peptide demonstrated that TIV vaccination elicited a more mature response and that serum antibody binding antibodies to rHA1 after vaccination strongly correlated with the MN titers against the 2009 pH1N1 vaccine strain. As previously demonstrated in ferrets [40], [41] and humans [42], these findings suggested that rHA1 oligomer-binding antibodies are involved in virus neutralization.

Our results provided evidence for modest antigenic drift in pH1N1 viruses from the southeastern United States in 2011. There were significant differences in vaccine-induced serum antibody neutralization in the vaccine strain 2009 pH1N1 as compared to the circulating 2011 pH1N1. Contemporaneously collected viruses from nationwide surveillance were rooted against the vaccine and other circulating strains to infer divergence in the HA surface protein.

Recruits arrive at the RTCs from regions across the United States, resulting in the potential seeding of diverse viruses. This was demonstrated in our study as isolates from four of six subclades (Groups 2, 3, 6, and 7) were found at Fort Jackson during the first 2 months of 2011. Interestingly, only one of these, Group 3, circulated widely during the outbreak in January 2011.

In the spring of 2009, distinct spatial heterogeneity existed within pH1N1 viruses, resulting in strong regional founder effects. During this first wave, multiple phylogenetically distinct pH1N1 clades emerged globally [43]. However, by the end of the second wave at the end of 2009, extensive viral migration and mixing resulted in the emergence of a single dominate viral lineage, in New York State [44]. The international profile of New York likely contributed to the seeding of this virus to regions across the world over the next 12–18 months [45], although other clades continue to circulate.

Analysis of the HA genome elucidated a number of mutations in 2011 pH1N1 viruses. The S183P mutation in the Fort Jackson viruses has been shown *in vitro* to inhibit the binding of the DFA monoclonal antibody from the WHO Influenza Detection Kit distributed in 2011 [46]. This mutation has been found in the 1918 pandemic influenza virus and shown to have increased virulence in mouse models [47]. The reversion mutation S84N, evident in viruses from Fort Jackson, has been associated with decreased antigenic responses [48]. These studies provide possible mechanisms underlying immune evasion in the pH1N1 viruses from the Fort Jackson region in 2011.

The outbreak of pH1N1 in vaccinated recruits occurred during a time when influenza A/H3N2 and B viruses and multiple subclades of A/pH1N1 circulated in the Fort Jackson. However, only one (Group 3) was evident during the outbreak. This circulating virus had important HA mutations that mediate antibody binding. Moderate antigenic divergence between circulating and vaccine influenza strains likely contributed to the outbreak of pH1N1 among recruits at Fort Jackson in the early weeks of 2011.

At Fort Jackson, the protective threshold was breached in LAIV vaccinees infected with antigenically divergent subclade 3 pH1N1 viruses. Increased TIV vaccination in the Fort Jackson recruit population could induce higher antibody titers and protective immunity. We speculate that TIV vaccination would increase overall vaccine effectiveness, thereby providing a herd immunity effect across the population.

The effectiveness of seasonal influenza vaccines varies by season. The risk of periodic influenza epidemics as a result of antigenic drift may best be ameliorated through the development of a universal influenza vaccine [49] and/or therapeutics that address issues of antiviral resistance, such as multi-drug combinational therapy [50]. Our work accentuates the need for intense surveillance tied to timely virus characterization and agile production of vaccines and therapeutics in response to ever-adapting influenza viruses.

## Materials and Methods

### Influenza Vaccination

Recruits undergoing basic combat training at Fort Jackson were vaccinated intranasally prior to the first week of training with the LAIV FluMist (MedImmune, LLC, Gaithersburg, MD) per manufacturer's instructions. Each 0.2 prefilled dose contained 10^6.5–7.5^ fluorescent focus units of live attenuated influenza virus reassortants of each of the three strains recommended by WHO for the 2010–2011 season: A/CA/7/2009 (H1N1), A/Perth/16/2009 (H3N2), and B/Brisbane/60/2008. Prior to the first week of recruit training at MCRD-PI, female recruits were vaccinated with the TIV influenza vaccine AFLURIA (CSL Biotherapies, King of Prussia, PA) per manufacturer's instructions. The AFLURIA influenza vaccine was standardized for the 2010–2011 influenza season and formulated to contain 45 mcg HA per 0.5 mL dose in the recommended ratio of 15 mcg HA for each of the three influenza strains recommended for the 2010–2011 Northern Hemisphere influenza season. Male recruits at MCRD-PI were vaccinated with the MedImmune LAIV FluMist, as previously described. Recruits at Cape May were vaccinated with the Fluzone TIV (Sanofi Pasteur, Inc., Swiftwater, PA) per manufacturer's instructions. Each 0.5-mL dose of Fluzone contained a total of 45 mcg of influenza virus HA equally distributed among the three components of the 2010–2011 influenza vaccine.

### Ethics Statement

The proposal for serologic draw was reviewed and approved as a public health response, non-research activity by the U.S. Army Public Health Command Public Health Review Board, the Institutional Review Board (IRB) at the Naval Health Research Center (NHRC), and the Armed Forces Health Surveillance Center. Participation for blood draw was voluntary, and consent obtained verbally per observation by local investigators. Per the proposal approved by the NHRC IRB, all samples were de-identified. Concurrently, throat and nasal swabs were collected under ongoing NHRC protocol NHRC.1999.0002 approved by the NHRC IRB from recruits who presented with an FRI and consented, in writing, to be swabbed. This project has been conducted in compliance with all applicable federal regulations governing the protection of human subjects in research.

### Sample Collection

The serosurvey was conducted by systematically enrolling recruits 4–5 week's post-influenza vaccination. Recruits completed a short case report form (CRF) that included information on previous vaccination, age, sex, and signs and symptoms. Influenza vaccination history (date, vaccine type) was abstracted from medical records at each training center. Blood was drawn in 10-ml serum separator tubes (BD Biosciences, Franklin Lakes, NJ) and allowed to clot for 30 minutes. Tubes were centrifuged for 10 minutes, and then frozen at between −20°C and −80°C prior to shipment to NHRC for analysis. Baseline sera were provided from the DoDSR, which maintains serum specimens collected from service members for periodic HIV testing and operationally required pre- and post-deployment blood draws [51]. Baseline samples were shipped to NHRC on dry ice.

Concurrently, throat and nasal swabs were collected from recruits who presented with an FRI. At RTCs, denominator data for all FRIs are collected and 10–20 patients are randomly selected for sampling each week throughout the year. FRI was defined by fever (>38.0°C), sore throat, and/or cough. Enrollees provided a CRF containing demographic and medical history data. Swabs were collected in universal transport medium (Copan Diagnostics, Inc., Murrieta, CA), stored at 4°C, and then shipped to NHRC for real-time RT-PCR (rRT-PCR), viral isolation, and genetic characterization.

### Virus Propagation and Microneutralization Assay

Influenza viruses were propagated in MDCK cells to high titer, and TCID_50_ determined using the Reed-Muench method. Negative and positive control sera for A/CA/7/2009 (2009 pH1N1) and A/Perth/16/2009 (H3N2) were obtained from the 2010–2011 WHO Influenza Reagent Kit. Positive control ferret antisera for A/CA/17/2011 (2011 pH1N1) were provided by CDC, Atlanta, Georgia. All control sera were treated with receptor-destroying enzyme (RDE), heat inactivated at 56°C for 30 minutes, and then diluted to a 1∶10 concentration. Serum antibody MN assays were performed according to described procedures [52], [53]_ENREF_25. Briefly, serum was inactivated at 56°C for 30 minutes and then 2-fold serum dilutions made in a range of 1∶5 to 1∶640. Positive control serum was serially diluted to 1∶1280; negative control was initially undiluted and then serially diluted 2-fold. After sera (both samples and controls) were serially diluted, 50 µl of working dilution (200 TCID_50_/50 µl) was added to the wells. Cell controls were included in each plate for data analysis. A/CA/7/2009 H1N1 (2009 pH1N1), A/Perth/16/2009 H3N2 (H3N2), and A/CA/17/2011 H1N1 (2011 pH1N1) were propagated in MDCK cells. After a 1-hour incubation at 37°C in 5% CO_2_, 30 ml of virus growth medium (Dulbecco's Modified Eagle's Medium containing 0.25% bovine serum albumin, 25 mM HEPES buffer, 100 U/ml penicillin, 100 µg/ml streptomycin, and 3 µg/ml TPCK-trypsin) was added and flasks incubated for 16 hours at 37°C in 5% CO_2_. MDCK flasks at 75–95% confluence were harvested and cell suspensions combined and centrifuged at 1500 rpm for 5 minutes. The culture supernatant was replaced with 15 ml of fresh virus growth medium and the cultures incubated 18–24 hours or until cells exhibited approximately 50% CPE and supernatants had a hemagglutination activity of at least 32 hemagglutination units using a 0.5% suspension of turkey erythrocytes. Following an overnight incubation, plates were decanted and washed once with 200 µl sterile PBS. Anti-influenza A nucleoprotein was added to fixed plates at a 1∶1000 dilution and incubated at room temperature for 1 hour. After washing, goat anti-IgG conjugated horseradish peroxidase at a 1∶2000 dilution was added for 1 hour. Absorbance in washed and blocked plates was read at 490 nm wavelength. MN titers were expressed as reciprocal of the highest dilution of serum that gave 50% neutralization. For calculation of geometric mean titer (GMT) estimates, a titer of <10 was assigned a value of 5. Seroprotection was determined by MN as the percentage of serum titers ≥40. Seroconversion rates were defined as the percentage of vaccine recipients whose serum MN titers increased by at least 4-fold after vaccination. A *p* value <0.05 was considered significant.

### Hemagglutination Inhibition Assay

The HI assays were performed according to the WHO protocol for serological diagnosis of influenza virus infection using standardized 0.75% guinea pig red blood cells (GPRBC). Serum samples were treated with RDEs overnight at 37°C, and heat inactivated the following day. Serum samples, including negative and positive controls, were initially diluted 1∶10 with PBS and then serially diluted 2-fold from 1∶10 to 1∶1280. Influenza viruses were adjusted to contain 4 HA units/25 µl and added to wells. GPRBC were added to all wells, and incubated at room temperature for 1 hour. Antibody titer for the particular virus was determined as the highest serum dilution showing complete inhibition for each serum sample tested.

### Statistical Analyses of MN and HI Data

Differences between groups were examined for statistical significance using Student's t-test. An unadjusted *p* value <0.05 was considered significant. For all MN and HI testing, each sample was tested in duplicate with the requirement that both results must agree to within one dilution. A titer of <10 was assigned a value of 5 for calculation purposes. GMTs of the replicates were used to estimate pre- and post-vaccination titers and the associated fold change.

Population pre- and post-vaccination GMTs were calculated for the three viruses (2009 pH1N1, 2011 pH1N1, H3N2) for each vaccination type (TIV and LAIV). Additionally, adjusted geometric mean fold change was estimated for each virus and vaccination type, after adjustment for starting titer as previously described [54]. Confidence intervals of all means were calculated using the least squares method, allowing us to evaluate the significance of the differences between vaccine type and virus strains.

Linear regression was used to evaluate differences in the geometric increases in HI and MN titers for different viruses and different types of vaccination. Logistic regression allowed differences in the odds of a 4-fold rise in titer to be evaluated between vaccination types and virus strains while adjusting for pre-vaccine levels. For this comparison, the rate of 4-fold rise in titer against H3N2 was used as a reference.

### Binding to rHA1 Proteins and Antibody Isotyping by Surface Plasmon Resonance

Steady-state equilibrium binding of post-immunization 2009 pH1N1 human vaccine sera was monitored at 25°C using a ProteOn SPR biosensor (Bio-Rad Laboratories, Inc., Hercules, CA), as previously described [55]. The 2009 pH1N1-rHA1 proteins were coupled to a GLC sensor chip with amine at 500 resonance units (RU) in the test flow cells. Samples of 60 µl of freshly prepared sera at 10-fold dilutions were injected at a flow rate of 30 µl/min (120-second contact time) for association, and dissociation was performed over a 600-second interval (at a flow rate of 30 µl/min). Responses from the protein surface were corrected for the response from a mock surface and for responses from a separate, buffer-only injection. MAb 2D7 (anti-CCR5) was used as a negative control in these experiments. Binding kinetics for the selected human vaccine sera and data analyses were calculated using Bio-Rad ProteOn manager software (version 3). For isotyping of the human serum antibodies bound to the rHA1 coupled chip, anti-human isotyping antibodies for human IgA, IgG, and IgM were injected onto the chip following the human sera interaction with coupled antigen on the GLC chip.

### Affinity Measurements by Surface Plasmon Resonance

Steady-state equilibrium binding of pre- and post-H1N1 human vaccine sera was monitored at 25°C using a ProteOn SPR biosensor (Bio-Rad). Antibody off-rate constants, which describe the stability of the complex (i.e., the fraction of complexes decaying per second), were determined directly from plasma sample interaction with properly folded, pH1N1-functional HA1 globular domain and HA2 stalk domain proteins [40] during the dissociation phase. They were calculated using the Bio-Rad ProteOn manager software for the heterogeneous sample model, as previously described [56]. To improve measurements, the off-rate constants were determined from two independent SPR runs. Differences between groups were examined for statistical significance using Student's t-test. An unadjusted *p* value <0.05 was considered significant.

### Real-Time Reverse Transcriptase Polymerase Chain Reaction Amplification

Ribonucleic acid was extracted from combined throat and nasal swabs using the QIAamp RNA Mini Kit (Qiagen, Valencia, CA) following manufacturer's instruction. rRT-PCR assays were used to detect influenza A and B viruses and to subtype A viruses as either H1, pH1, or H3, as previously described [57]. One-step rRT-PCR was performed in a final volume of 25 µL, which contained 5 µl of extracted RNA, 12.5 µL of buffer mix, and 0.5 µL SuperScript III Platinum Taq enzyme (Invitrogen, Carlsbad, CA), 0.8 µM for each primer, and 0.2 µM of probe. A 7500 Fast DX Real-Time PCR System (Applied Biosystems Inc., Foster City, CA) was used for rRT-PCR reactions. The thermocycling parameters for targets consisted of 50°C for 30 minutes, 95°C for 2 minutes, and 45 cycles with 95°C for 15 seconds, and 55°C for 30 seconds.

### Viral Isolation

Virus isolation was performed in MDCK cells or R-Mix Too Shell Vials (Diagnostic Hybrids, Inc. [DHI], Athens OH). In shell vials, medium was aspirated and 1 mL of R-Mix Refeed media (DHI) containing 100 units/mL penicillin, 100 ug/mL streptomycin, and trypsin (bovine origin) at 1.33 ug/mL, was added. Vials were inoculated with 0.2 mL of swab extract and then centrifuged at 2100 rpm (RT) for 1 hour. After centrifugation, vials were incubated for 48 hours, washed with sterile PBS twice, and fixed in acetone. Immunofluorescence assays were conducted using D3 Ultra DFA Respiratory Virus Screening Reagent (DHI). Viruses were replicated and amplified in MDCK flasks, at 80–90% confluency. Upon CPE approximately 75% of the susceptible cells were harvested and centrifuged at 2000 rpm, at 4°C, for 5 minutes. The TCID_50_ for each of the viruses were calculated to obtain 200 TCID_50_/50 µL.

### HA1 Sequencing

Primers for sequencing influenza HA protein were provided by the CDC and are available upon request. An initial amplicon was produced with a paired primer set in a 25 uL aqueous reaction containing 1× Colorless GoTaq Flexi Reaction Buffer (Promega Corporation, Madison, WI), 0.2 mM each dNTP (Promega), 1.5 mM MgCl2 (Promega), 0.6 uM concentration of each primer, 0.5 U GoTaq DNA Polymerase (Promega), and 5 uL of template. Products were mixed 5∶1 with loading dye (Sigma-Aldrich, St. Louis, MO) and run for 90 min at 125 V on 2% agarose (Bio-Rad, Hercules, CA) gels with ethidium bromide (Sigma-Aldrich). Cycling conditions were an initial 60 seconds at 96°C followed by 25 cycles of 10 seconds, 96°C; 5 seconds, 50°C; 4 minutes, 60°C, and a final hold at 40°C. Gels were visualized in an ultraviolet light box. Amplicons were excised and purified using the QIAquick gel extraction kit (Qiagen) according to manufacturer's instructions. Amplicons were then used as template in sequencing reactions containing 1× BigDye Terminator v3.1 Buffer (Applied Biosystems, Foster City, CA), 1 uL of BigDye Terminator Ready Reaction Mix v. 3.1, 0.2 uM forward or reverse primer, and 5 uL of amplicon in a 20 uL aqueous reaction. Reactions were performed on an iCycler PCR machine (Bio-Rad). Sequencing products were purified using Performa DTR Gel Filtration Cartridges (Edge BioSystems, Gaithersburg, MD) and sequenced on a 3130 Genetic Analyzer (Applied Biosystems).

### Whole Genome Analysis

Sequencing, genome assembly, and closure reactions were performed as described [58]. Briefly, influenza multiplex RT-PCR products were randomly amplified and prepared for next-generation sequencing using a sequence-independent single-primer amplification (SISPA) method as previously described [59]. Amplified viral DNA was denatured in the presence of dimethyl sulfoxide and a chimeric oligonucleotide containing a known bar code 22-nt sequence followed by a 3′ random hexamer. A Klenow reaction was prepared with the denatured DNA template. The resulting cDNA was randomly amplified by PCR using AccuPrime Taq at 35 cycles (denaturation: 30 seconds, 94°C; annealing: 30 seconds, 55°C; extension: 48 seconds, 68°C). PCRs were conducted using primers corresponding to the known 22-nt bar-code sequence from the oligonucleotide utilized in the previous Klenow step. SISPA products were normalized and pooled into a single reaction that was subsequently purified. This sample was gel purified to select for SISPA products <800 bp in size. Identical aliquots were then submitted for sequencing with both 454 (Clinical Genomics, Australia) and Illumina (DNASTAR, Inc., Madison, WI) sequencing technologies.
